# Phycocyanin Inhibits Tumorigenic Potential of Pancreatic Cancer Cells: Role of Apoptosis and Autophagy

**DOI:** 10.1038/srep34564

**Published:** 2016-10-03

**Authors:** Gaoyong Liao, Bing Gao, Yingnv Gao, Xuegan Yang, Xiaodong Cheng, Yu Ou

**Affiliations:** 1 School of Life Science & Technology, China Pharmaceutical University, Nanjing, China; 2 Department of Integrative Biology and Pharmacology, The University of Texas Health Science Center, Houston, USA

## Abstract

Pancreatic adenocarcinoma (PDA) is one of the most lethal human malignancies, and unresponsive to current chemotherapies. Here we investigate the therapeutic potential of phycocyanin as an anti-PDA agent *in vivo* and *in vitro*. Phycocyanin, a natural product purified from Spirulina, effectively inhibits the pancreatic cancer cell proliferation *in vitro* and xenograft tumor growth *in vivo*. Phycocyanin induces G2/M cell cycle arrest, apoptotic and autophagic cell death in PANC-1 cells. Inhibition of autophagy by targeting Beclin 1 using siRNA significantly suppresses cell growth inhibition and death induced by phycocyanin, whereas inhibition of both autophagy and apoptosis rescues phycocyanin-mediated cell death. Mechanistically, cell death induced by phycocyanin is the result of cross-talk among the MAPK, Akt/mTOR/p70S6K and NF-κB pathways. Phycocyanin is able to induce apoptosis of PANC-1 cell by activating p38 and JNK signaling pathways while inhibiting Erk pathway. On the other hand, phycocyanin promotes autophagic cell death by inhibiting PI3/Akt/mTOR signaling pathways. Furthermore, phycocyanin promotes the activation and nuclear translocation of NF-κB, which plays an important role in balancing phycocyanin-mediated apoptosis and autosis. In conclusion, our studies demonstrate that phycocyanin exerts anti-pancreatic cancer activity by inducing apoptotic and autophagic cell death, thereby identifying phycocyanin as a promising anti-pancreatic cancer agent.

Pancreatic cancer is one of the gastrointestinal tumors with the poorest prognosis. In the United States, pancreatic adenocarcinoma (PDA) is the fourth leading cause of cancer-related death^1^. Current major treatment modalities for pancreatic cancer includes surgical resection, radiotherapy and chemotherapy. However, the 5 year survival rate for pancreatic cancer is lower than 4% due to its aggressive nature, prone to metastasis and resistance to most chemotherapies^2^,^3^. Therefore, there is an urgent need to further understand the pathobiology of PDA and to develop new and effective therapeutic strategies.

Phycocyanin, one of the major pigment constituents of Spirulina microalgae, exists as a mixture of the oligomers composed of the alpha subunit and the beta subunit containing chromophore^4^. Phycocyanin is used in many countries as a dietary supplement whose nutritional values have been very well documented^5^,^6^.

Accumulating evidence shows that phycocyanin has a potent anticancer effect both *in vitro* and *in vivo* on a variety of cancer cell types, such as lung cancer^7^, colon cancer^8^, breast cancer^9^ and bone marrow cancer^10^. In addition, administration of phycocyanin at the high doses from 0.25 to 5.0 g/kg body weight (w/w) does not induce noticeable symptoms of toxicity nor mortality in animals^11^. These studies suggest a therapeutic potential of phycocyanin in cancer treatment.

Mechanistically, phycocyanin exerts its anti-cancer effect by modulating apoptosis and cell proliferation. Phycocyanin has been demonstrated to induce apoptosis in tumor cells through the production of ROS and down-regulating the expression of Bcl-2, a well-known anti-apoptotic molecule^12^, as well as through inducing cytochrome c release from mitochondria into the cytosol and PARP cleavage^13^. Phycocyanin can also induced apoptotic cell death by upregulation of Caspase 3 and Caspase 8 activities^14^. Phycocyanin’s anti-cell proliferative effects are mediated by inactivation of BCR-ABL signaling and the downstream PI3K/Akt pathway^15^.

Accumulating evidence has demonstrated that targeting autophagy is a promising and alternative strategy for developing anti-cancer therapy^16^. Besides its well-known pro-survival role, autophagy represents a double-edged sword and may also contribute to cell damage^17^,^18^. In particular, previous reports reveal the existence of a complex crosstalk between autophagy and apoptosis, and the two processes are usually induced by the same stimuli and share similar effectors and regulators^19^,^20^,^21^. These studies suggest that it is possible to develop anti-cancer therapeutic strategies by synergistically modulating autophagy and apoptosis processes.

To date, neither the role of phycocyanin in pancreatic cancer nor the effect of phycocyanin on autophagy has been investigated. In the present study, we investigate the anti-pancreatic cancer effect of phycocyanin on human PDA *in vitro* and *in vivo*. We find that phycocyanin efficiently inhibits pancreatic cancer cell growth by inducing apoptotic and autophagic cell death. Furthermore, we uncover the signaling pathways involved in phycocyanin-mediated autophagy and apoptosis. To the best of our knowledge, this is the first study to demonstrate that phycocyanin induces autophagy in pancreatic cancer cells, which is regulated by inhibition of the Akt/mTOR/p70S6K pathway and stimulation of the NF-κB pathway.

## Results

### Phycocyanin inhibits cell viability in tumor cells and has little effect on normal cell line

**Figure 1:**
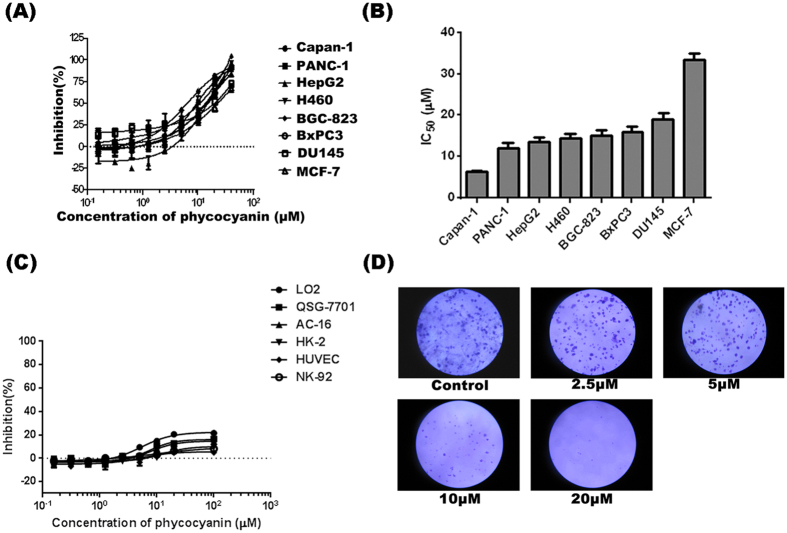
Phycocyanin inhibits cell viability in tumor cells and has little effect in normal cells. (**A**) Growth curves of capan-1, PANC-1, BxPC3, BGC-823, HepG2, H460, DU145 and MCF-7 cancer cell line in the presence of various concentration phycocyanin. **(B)** IC50 values for growth inhibition of different cancer cell lines including capan-1, PANC-1, BxPC3, BGC-823, HepG2, H460, DU145 and MCF-7 by phycocyanin. Different cancer cells were treated for 72 h with increasing concentrations of phycocyanin. Results are presented as the mean ± SD of at least three independent experiments, each performed in triplicate. (**C**) Growth curves of LO2, QSG-7701, AC-16, HK-2, HUVEC and NK-92 cell line in the presence of various concentration phycocyanin. (**D**) The suppressive effect of phycocyanin on the colony formation of PANC-1 cells.

To investigate the anti-cancer potential of phycocyanin, we monitored the effect of phycocyanin on cell viability of a panel human cancer cell lines using a metabolic activity-based CellTiter-Glo^®^ luminescent assay. As shown in Fig. 1A, phycocyanin dose-dependently inhibited the growth of a broad spectrum of cancer cell lines, including Capan-1, PANC-1, HepG2, H460, BGC-823, DU145 and MCF-7. Phycocyanin showed particularly good inhibitory activity towards several pancreatic cancer cell lines, Capan-1, PANC-1 and BxPC3 with apparent IC50 of 6.2 ± 0.2, 12.2 ± 1.2 and 15.1 ± 1.3 μM, respectively (Fig. 1B). To investigate the possible potential toxicity of phycocyanin, we also monitored the effect of phycocyanin on cell viability of a panel normal cell lines including LO2, QSG-7701, AC-16, HK-2, HUVEC and NK-92. As shown in Fig. 1C, phycocyanin has almost no inhibitory effect on normal cells.

The growth inhibitory effect of phycocyanin on PANC-1 cells was also time-dependent, became more potent as time passed (Fig. S1A). In the presence of 10 μM phycocyanin, PANC-1 cells displayed more elongated cell morphologies, and eventually detached from the culture dish after 72 h incubation with phycocyanin (Fig. S1B). Colony formation assay was performed to investigate the effect of phycocyanin on tumorigenic potential of PANC-1 cells. Colony-forming ability of PANC-1 cells was dose-dependently reduced after exposure to phycocyanin and almost completed abolished at the concentration of 20 μM (Fig. 1D). These results indicate that phycocyanin inhibits the tumorigenicity of PANC-1 *in vitro*.

### Phycocyanin inhibits pancreatic cancer tumor growth *in vivo*

**Figure 2:**
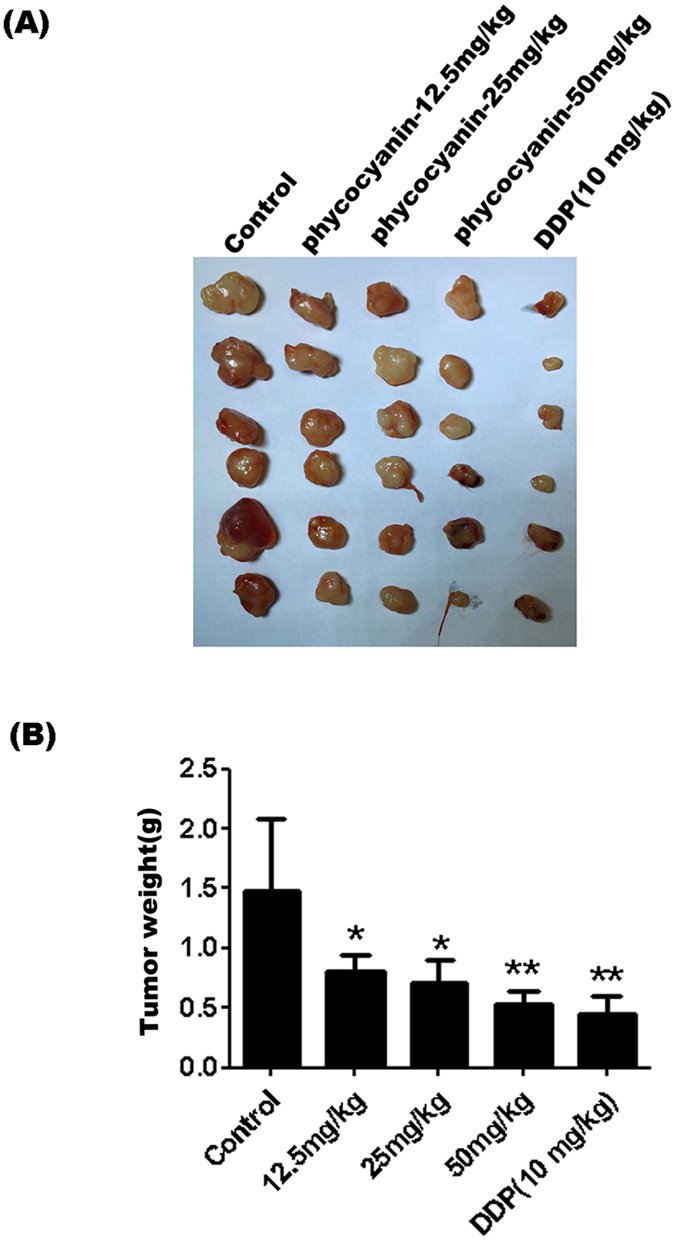
Phycocyanin inhibits pancreatic cancer tumor growth *in vivo*. (**A**) Representative PANC-1 xenograft tumors from mice treated with vehicle, DDP (10 mg/kg) and phycocyanin (12.5-50 mg/kg). (**B**) Tumor weights of xenograft tumors from mice treated with vehicle, DDP (10 mg/kg) and phycocyanin (12.5–50 mg/kg). Data were presented as the mean ± SD, n = 6 for each group, *P < 0.05; **P < 0.01.

PANC-1 based tumor xenograft model was used to evaluate the antitumor effect of phycocyanin *in vivo*. As shown in Fig. 2A, treatment with 12.5, 25, and 50 mg/kg phycocyanin dose-dependently inhibited tumor growth. At the end of the experiment, the tumor weights were significantly reduced in the groups treated with 12.5, 25, and 50 mg/kg phycocyanin or 10 mg/kg DDP with corresponding tumor inhibition rates at 45.62 ± 10.52%, 51.51 ± 12.31%, 64.25 ± 7.82%, and 69.96 ± 10.39% respectively (Fig. 2B, Fig. S2A). The tumor inhibition potency of phycocyanin at 50 mg/kg was similar to that of the positive control (10 mg/kg DDP). On the other hand, while the mice under 10 mg/kg DDP treatment exhibited significant body weight loss, phycocyanin treatment had no effect on mice body weight as compared to vehicle control (Fig. S2B). These observations are consistent with our previous findings that phycocyanin administration does not lead to hepatic toxicity and protects against carbon tetrachloride-induced hepatocyte damage *in vitro* and *in vivo*^22^. Taken together, these results suggest that phycocyanin processes notable anti-pancreatic tumor activity without obvious toxicity.

### Phycocyanin blocks G2/M cell cycle progression and induces caspase 3-independent cell death in PANC-1 cells

**Figure 3:**
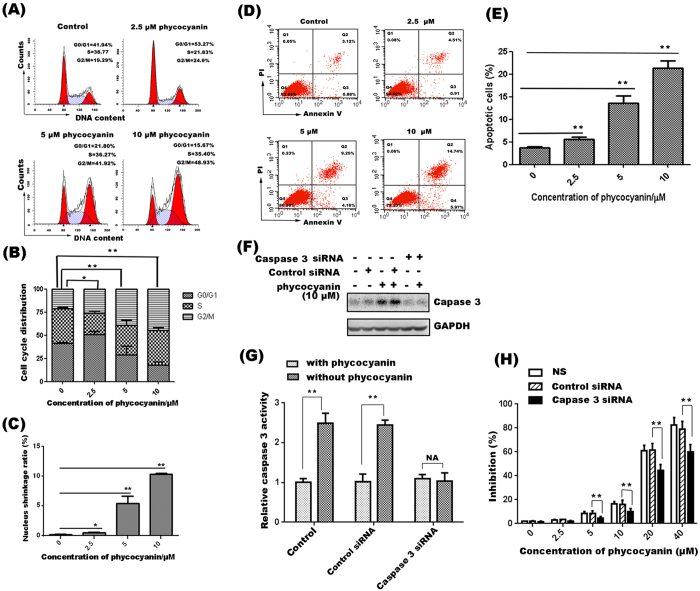
Phycocyanin blocks G2/M cell cycle progression and induces caspase 3 independent cell death in PANC-1 cells. (**A**) The effect of phycocyanin on the cell cycle distribution of PANC-1 cells. PANC-1 cells treated with indicated concentrations of phycocyanin for 72 h were fixed, stained and analyzed for DNA content by FACS. (**B**) The distribution and percentage of cells in G0/G1, S and G2/M phase of the cell cycle were calculated and plotted. The statistically significant differences between the treated cells and the control group were indicated by *P < 0.05, **P < 0.01. (**C**) Nuclear shrinkage ratio was analyzed with cell scoring model of High Content Screening system software. The data shown were representative of three independent experiments. The statistically significant differences between the treated cells and the control group were indicated by *P < 0.05, **P < 0.01. (**D**) Flow cytometry analysis of PANC-1 cell apoptosis induced by phycocyanin for 72 h. (**E**) Following treatment with phycocyanin, early apoptotic cell population with Annexin V-positive and late apoptotic cell population with PI- positive PANC-1 cells increased in a dose-dependent manner. Mean ± SD of three assays. *P < 0.05, **P < 0.01, versus control. (**F**) Knockdown of caspase 3 by siRNA pool for 24 h followed by 10 μM phycocyanin treatment for 48 h decreases capase 3 expression in PANC-1 cells when compared to cells treated with lipofectamine alone (NS) or non-silencing siRNA (Control siRNA) and 10 μM phycocyanin. (**G**) Caspase 3 siRNA inhibited the activation of caspase 3 in PANC-1 cells induced by phycocyanin. Knockdown of caspase 3 by siRNA pool for 24 h followed by 10 μM phycocyanin treatment for 72 h decreases caspase 3 activation. The bars represent mean ± SD, n = 3, **P < 0.01, NA: No significance (t- test). (**H**) Dose-dependent growth cell inhibition by phycocyaninon in PANC-1 cells transfected with caspase 3 or control siRNA. PANC-1 cells transfected with caspase 3 or control siRNA were treated with indicated concentrations of phycocyanin for 48 h. Data were means ± SD of three independent experiments. *P < 0.05, **P < 0.01 (*t-test*).

To determine potential cellular mechanisms of growth inhibition effects of phycocyanin, we tested the effects of phycocyanin on cell cycle progression of PANC-1 cells by performing flow cytometric analyses of the cells after 72 h treatment with different concentrations of phycocyanin. The results showed that phycocyanin treatment led to a significant increase in G2/M cell population and a decrease in G0/G1 phase population in a dose-dependent manner (Fig. 3A,B). Phycocyanin induced cell cycle arrest was accompanied by a modest and dose-dependent cell apoptosis in PANC-1 cells (Fig. 3C–E). To determine if phycocyanin-induced cell death was mainly caused by apoptosis, we further examined the role of phycocyanin in the PANC-1 cells with the apoptotic–specific gene caspase 3 silenced by siRNA. The results showed that caspase 3 was successfully knocked down by its siRNA (Fig. 3F,G). Phycocyanin-mediated growth inhibition was significantly reduced by not completely reversed as compared to that of the NS group and control siRNA group (Fig. 3H). These data suggested that cell death induced by phycocyanin in PANC-1 cells was only partially dependent on caspase 3 activation, and additional pathways are also involved.

### Phycocyanin induces significant autophagy in PANC-1 Cells

**Figure 4:**
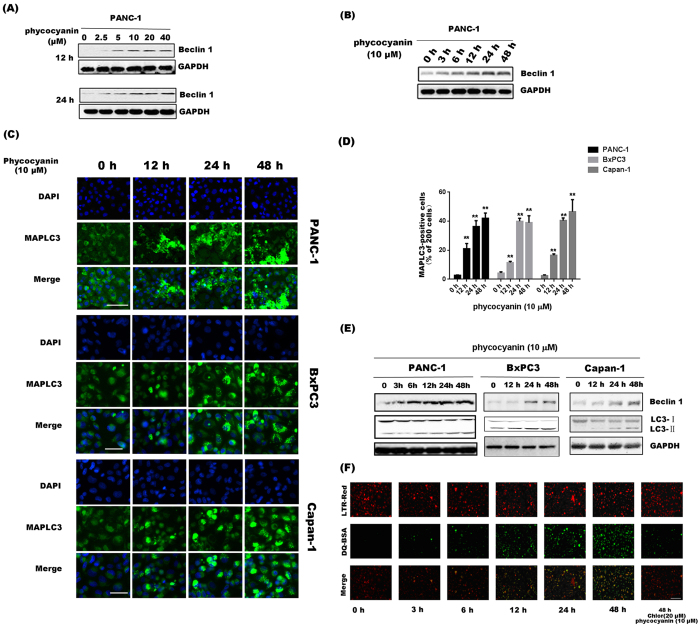
Phycocyanin induces significant autosis in PANC-1 Cells. (**A**) Phycocyanin induced dose-dependent Beclin 1 protein expression in PANC-1 cells. Western blot analysis of Beclin 1 expression after 0, 2.5, 5, 10, 20 and 40 μM phycocyanin treatment for 12 h and 24 h. (**B**) Phycocyanin induced time-dependent Beclin 1 protein expression in PANC-1 cells. Western blot analysis of Beclin 1 expression after 3, 6, 12, 24 and 48 h of treatment with 10 μM phycocyanin. (**C**) Fluorescence images of pancreatic cancer cells showing endogenous MAP-LC3 levels at different time points after phycocyanin treatment. Nuclei (blue) were labeled by DAPI. MAP-LC3 expression (green) was detected using an anti-MAP-LC3 polyclonal antibody. Goat anti-rabbit IgG/FITC were used as secondary antibody. Fluorescence images were obtained under a high content imaging system. Bar = 100 μm. (**D**) Quantification of the percentage of cells with focal MAP-LC3 at the indicated time after phycocyanin treatment. Error bars correspond to SD of three repeated wells, counting 200 cells each. *P < 0.05; **P < 0.01, 0 h versus phycocyanin-treated cells. (**E**) Effect of phycocyanin on expression of LC3 lipidation and Beclin 1 levels in pancreatic cancer cells were analyzed by Western blotting. Cells were treated with 10 μM phycocyanin for the indicated times. (**F**) Fluorescence visualization of lysosomal-dependent proteolysis upon cleavage and release of the fluorescent moiety of DQ-BSA (green) in phycocyanin-treated PANC-1 cells. Chloroquine is added to monitor DQ-BSA emission in cells with blocked lysosomal activity. Cells were simultaneously imaged in the presence of Lysotracker red (LTRred) to visualize the lysosomal compartment. Bar = 100 μm.

To investigate the alternative mechanism of cell death induced by phycocyanin, we investigated the effect of phycocyanin on Beclin 1, the mammalian orthologue of yeast Atg6 and a key regulator of autophagy^22^ and autosis^23^. Cellular Beclin 1 level was markedly increased in a time-and dose-dependent manner after phycocyanin treatment (Fig. 4A,B). We next assessed whether phycocyanin treatment induced the initiation of autophagy in pancreatic cancer cells by examining the cellular distribution of microtubule-associated protein 1 light chain 3 (LC3), a molecular marker of autophagosomes. Distinct punctate patterns of LC3 immunofluorescence, representing increased formation of autophagic vacuoles, were observed in cells treated with phycocyanin (10 μM) (Fig. 4C). The percentages of MAP-LC3-positive cells were increased significantly in different pancreatic cancer cells after treatment with 10 μM phycocyanin for indicated time (Fig. 4D). The formation of the punctate staining of LC3 induced by phycocyanin was accompanied by a parallel and time-dependent up-regulation of Beclin 1 and the conversion of LC3 from a soluble form (LC3-I) to the lipidated and autophagosome associated form (LC3-II) (Fig. 4E).

The autophagosomes undergo acidification after maturation and fusion with lysosomes so that their content is digested by lysosomal hydrolases at a late stage of autophagy. The ability to process DQ-BSA, a derivative of BSA whose green fluorescence is quenched unless cleaved by proteolyticenzymes, in lysosomal compartments was used to evaluate the progression of autophagy in response to phycocyanin treatment. A time-dependent enhancement of green fluorescence signals, co-localized with the lysosomal marker, lysotracker Red, was observed after phycocyanin treatment, indicating that DQ-BSA was efficiently cleaved in the presence of phycocyanin. On the other hand, co-treatment of the cell with a lysosomotropic agent chloroquine that prevents endosomal acidification, blocked the processing of DQ-BSA (Fig. 4F), and increased levels of the lysosomal marker lysosome-associated membrane glycoprotein 1 (LAMP-1) and cathepsin D, the predominant lysosomal aspartic protease, induced by phycocyanin treatment (Fig. S3).

### Inhibition of autophagy by Beclin 1 siRNA rescues phycocyanin-mediated cell death in PANC-1 cells

**Figure 5:**
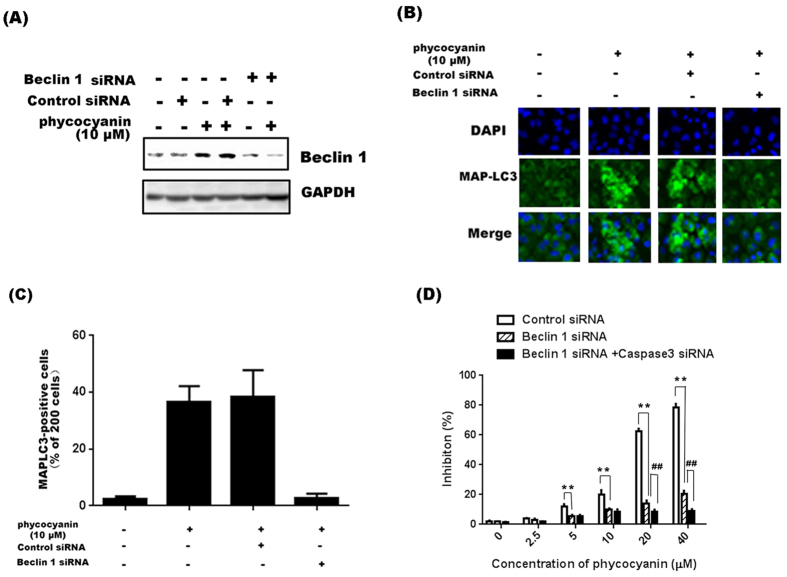
Inhibition of autophagy by Beclin 1 siRNA rescues phycocyanin-mediated cell death in PANC-1 cells. (**A**) Knockdown of Beclin 1 by siRNA pool for 24 h followed by 10 μM phycocyanin treatment for 48 h decreases Beclin 1 expression in PANC-1 cells when compared to cells treated with non-silencing siRNA (Control siRNA) and 10 μM phycocyanin. (**B**) Treatment of PANC-1 with 10 μM phycocyanin for 24 h following a knockdown of Beclin 1 gene with siRNA pool for 48 h shows a significant decrease in MAP-LC3 punctate pattern as compared to cells treated with non-silencing siRNA (control siRNA) or lipofectamine alone followed by phycocyanin. (**C**) Quantification of the percentage of cells with focal MAP-LC3 at the indicated conditions. Error bars correspond to SD of three repeated wells, counting 200 cells each. *P < 0.05; **P < 0.01, 0 h versus phycocyanin-treated cells. (**D**) PANC-1 cells treated with 10 μM phycocyanin for 48 h following Beclin 1 only and Beclin1 and caspase 3 dual silencing showed a significant decrease of cell viability inhibition when compared to cells treated with control siRNA alone along with phycocyanin. The bars represent mean ± SD, n = 3, **control siRNA group *vs* Beclin 1 siRNA group, *P* < 0.01; ^##^“Beclin 1 siRNA” group *vs* “Beclin 1 siRNA + Caspase 3 siRNA” group, *P* < 0.01 (t test).

To determine if autophagy plays a role in phycocyanin-mediated growth inhibition and cell death, the expression of the autophagy-related protein Beclin 1 was silenced using Beclin 1 specific siRNA. As shown in Fig. 5A, Beclin 1 was successfully knocked down by its siRNA but not the non-silencing control siRNA. Moreover, focal MAP-LC3 induced by phycocyanin was significantly inhibited by Beclin 1 siRNA (Fig. 5B,C). Importantly, silencing of Beclin 1 significantly suppressed phycocyanin-mediated growth inhibition of PANC-1 cells, and concomitantly administration of both Beclin1 and caspase3 siRNAs led an almost complete rescue of phycocyanin-induced cell growth inhibition (Fig. 5D). These results suggested that autophagy plays a major role in phycocyanin-induced PANC-1 cell death.

### Involvement of the MAPK, PI3K/Akt/mTOR and NF-κB pathway in phycocyanin-induced cell death

**Figure 6:**
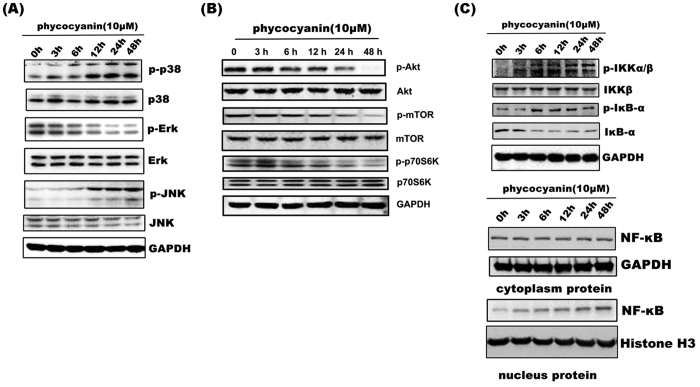
Involvement of the MAPK, PI3K/Akt/mTOR and NF-κB pathway in phycocyanin-induced cell death. (**A**) Effect of phycocyanin on the MAPK signaling in PANC-1 cells. After treatments, the levels of Erk1/2, JNK and p38 MAPK and their phosphorylated forms were analyzed by Western blotting with indicated antibodies. Results shown were representative of at least three independent experiments. (**B**) Western blot analysis of pAkt, total Akt, p-mTOR, total mTOR, p-p70S6K and total p-p70S6K in PANC-1 cells. GAPDH was shown as a loading control. Data are representative from three independent experiments. (**C**) Phycocyanin time dependently activiated NF-κB signaling pathway and induced the nuclear localization of NF-κB. After treatments, the levels of IKKβ and IκB-α and their phosphorylated forms were analyzed by Western blotting with indicated antibodies. Cytosolic and nuclear proteins were subjected to 10% SDS-polyacrylamide gels followed by western blot analysis for NF-κB p65. The results were representative of three independent experiments.

To determine the molecular mechanism of phycocyanin-mediated cell death, we investigated phycocyanin’s effects on MAPK and PI3K/Akt/mTOR signaling pathways. While the expression of total JNK, p38 and Erk was not significantly affected by phycocyanin treatment, phycocyanin increased the levels of p-JNK and p-p38 and decreased the level of p-Erk in a time-dependent manner (Fig. 6A). Similarly, phycocyanin suppressed p-Akt (Ser473), p-mTOR (Ser2448) and p-p70S6K in a time-dependent manner, while the expression of total Akt, mTOR and p70S6K did not change significantly (Fig. 6B).

Our study showed that phycocyanin induced the expression of Beclin 1 (Fig. 4A,B). Considering that NF-κB is an important transcription factor for Beclin 1^24^, we determined if phycocyanin activated the NF-κB pathway. Phycocyanin treatment led to an increased p-IKKβ level without obvious changes in the total IKKβ. On the other hand, p-IκB-α was upregulated but IκB-α was downregulated (Fig. 6C). Meanwhile, phycocyanin treatment time-dependently increased the nuclear fraction of NF-κB without affecting the cytoplasmic NF-κB (Fig. 6C).

### Phycocyanin induced apoptic cell death through MAPK pathway and induced autosis through NF-κB translocation

**Figure 7:**
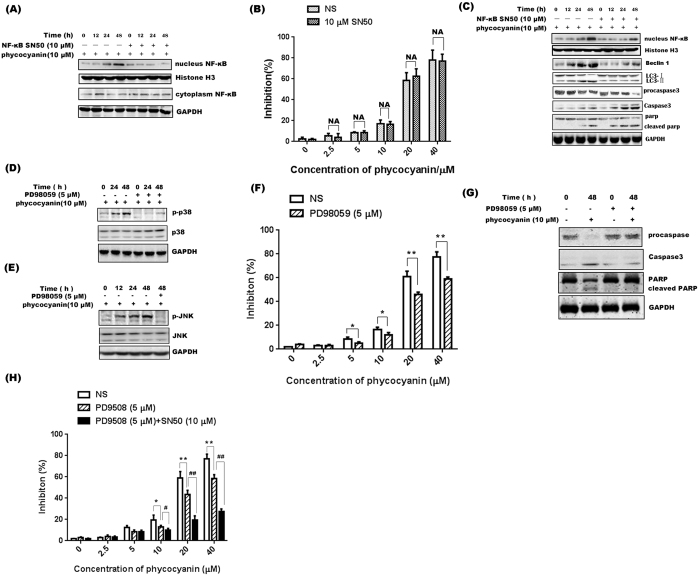
Effect of phycocyanin on cell viability and apoptosis marker expression in PANC-1 cells in the case that the MAPK and the NF-κB pathway are separately and simultaneously suppressed. (**A**) The nuclear localization of NF-κB after phycocyanin treatment was blocked by SN50. Cells were treated with 10 μM phycocyanin for different time with SN50 (10 μM). Cytosolic and nuclear proteins were used for western blot analysis using anti-NF-κB p65 antibody. (**B**) Effect of phycocyanin on PANC-1 cell viability treated with SN50. The data shown are representative of three independent experiments. The bars represented mean ± SD, n = 3. *P < 0.05, **P < 0.01, NA: No significance (t-test). (**C**) Western blot analysis of nucleus NF-κB, LC3 II/I, Beclin 1, procaspase, caspase and PARP expression in PANC-1 cells treated with 10 μM phycocyanin in absence or presence of SN50 (10 μM) for indicated time point. (**D**) Activation of p38 was effectively inhibited by the specific MEK inhibitor PD98059. (**E**) Activation of JNK was effectively inhibited by the specific MEK inhibitor PD98059. (**F**) Effect of phycocyanin on PANC-1 cell viability treated with PD98059. The data shown are representative of three independent experiments. The bars represented mean ± SD, n = 3, *P < 0.05, **P < 0.01, NA: No significance (t-test). (**G**) Western blot analysis of procaspase, caspase 3 and PARP expression in PANC-1 cells treated with 10 μM phycocyanin in absence or presence of PD98059 (5 μM) for 48 h. (**H**) Effect of phycocyanin on PANC-1 cell viability after inhibition of MAPK pathway alone and simultaneous inhibition of MAPK and NF-κB pathway. The bars represent mean ± SD, n = 3. Control group (NS) *vs* PD98059 group: *P < 0.05, **P < 0.01(t test); “PD98059” group *v*s “PD98059 + SN50” group: ^#^*P* < 0.05, ^##^*P* < 0.01 (t-test).

To further validate the involvement of the aforementioned signal pathways phycocyanin-mediated cell death, NF-κB SN50 (a NF-κB translocation inhibitor, SN50) and PD98059 (a special MEK inhibitor), were used to block the corresponding signaling pathway. SN50 significantly inhibited the translocation of NF-κB from cytoplasm to nucleus (Fig. 7A), but did not reduce the inhibition effect of phycocyanin on PANC-1 cells (Fig. 7B). In order to clarify the role of NF-κB translocation in the regulation of apoptosis and autophagy, related protein Beclin 1, MAP-LC3, capase3, and PARP were probed by Western Blotting analysis. As shown in Fig. 7C, SN50 inhibited the expression of Beclin 1 and conversion of LC3-I to LC3-II while significantly increased active caspase 3 and cleaved PARP were also observed. On the other hand, PD98059 successfully inhibited the activation of p38 and JNK (Fig. 7D,E), and significantly inhibited the phycocyanin-induced cell growth inhibition (Fig.7F). Consistent with these findings, PD98059 treatment significantly blocked phycocyanin-induced caspase 3 activation and PARP cleavage (Fig. 7G). Furthermore, combination treatment with PD98059 and SN50 further reduced the phycocyanin-induced PANC-1 cell death compared to MAPK inhibition alone (Fig. 7H). These results indicate that MAPK and NF-κB signaling pathways collaborate with each other and contribute directly to PANC-1 cell death induced by phycocyanin.

## Discussion

In this study, we have demonstrated that phycocyanin inhibits the growth of multiple cancer cell lines and effectively inhibited pancreatic cancer tumor growth in mouse xenograft tumor model. These results are consistent with previously studies showing that phycocyanin can suppress the growth of a variety of tumor cell lines^7^,^25^,^26^. The ability of phycocyanin to inhibit the growth of pancreatic cancer cells *in vitro* and *in vivo* is of particular interest as this is the first demonstration of phycocyanin’s activity against pancreatic cancer, an extremely aggressive and bad form of cancer with few effective therapeutic options. Previous studies suggest that phycocyanin exerts its anti-cancer activity by inducing cell apoptosis and cell cycle arrest^12^,^15^. Indeed, our results showed that phycocyanin blocked the G2/M cell cycle progression and induced apoptosis in PANC-1 cells. However, to our surprise, gene silencing of caspase 3 by caspase 3 siRNA was only marginally effective in suppressing phycocyanin-mediated growth inhibition and cell death. These results indicate that the mechanism of phycocyanin-mediated cell growth inhibition and cell death is complex and that other cellular processes in addition to apoptosis may also contribute to phycocyanin’s anticancer activity.

Although autophagy is designated as programmed cell death type II, whether autophagy actually promotes or protects cells from death remains controversial^27^. The role of autophagy on cell death is more likely pathway-specific and depending on how autophagy is induced^28^. In this study, we provided convincing evidence to show that phycocyanin induced autophagy in PANC-1 cells as phycocyanin treatment led to a time- and dose-dependent increase in expression of Beclin 1, the mammalian orthologue of yeast Atg6 that plays a central role in autophagy induction, and the formation of characteristic autophagosomes. Importantly, our study demonstrates that autophagy is responsible for phycocyanin-induced growth inhibition and death of PANC-1 cells as inhibition of autophagy by silencing Beclin 1 expression largely negates the growth inhibition effect imposed by phycocyanin. Furthermore, silencing both Beclin 1 and caspase 3 leads to an almost complete rescue of phycocyanin-mediated cell death. Our results are consistent with the notion that autophagy and apoptosis often co-exist, and maintain a balance with each other^29^.

To determine the molecular mechanisms and the signaling pathways that phycocyanin utilizes to induce cancer cell apoptosis and autophagy, we continue to explore the roles of the MAPK signaling pathways. Among the three subfamilies of MAPKs (JNK, p38 and Erk), the dynamic balance among growth factor-activated Erk and stress-activated JNK and p38 pathways may be critical in determining whether a cell survives or undergoes apoptosis^30^. It has been originally shown that Erks are essential for cell survival, whereas JNKs and p38-MAPKs were deemed stress responsive and thus involved in apoptosis^31^,^32^,^33^. Consistent with previous literature^34^,^35^, our findings that phycocyanin activated the JNK and p38 pathways while suppressed the Erk signaling suggest that MAPK signaling pathways play an important role in phycocyanin-induced apoptosis in cancer cells.

On the other hands, Mammalian target of rapamycin, mTOR, has been known as a key regulator of autophagy^36^. Inhibition of the mTOR pathway is consistently associated with triggering autophagy in cancer cells^37^,^38^. The protein kinase Akt activates mTOR via direct phosphorylation and inhibition of tuberous sclerosis complex 2 (TSC2), which is a negative regulator of mTOR^39^. Akt inhibition decreases mTOR activity and promotes autophagy. Several studies have also shown that Akt/mTOR/p70S6K pathway plays an important role in autophagy development for various cancer cells including liver cancer^40^, astric cancer^41^, pancreatic cancer^42^ and malignant glioma^28^. Our results revealed that phycocyanin inhibited Akt/mTOR/p70S6K signal pathway, which may contribute to phycocyanin-induced autophagy.

Recent studies demonstrate that despite the marked differences between apoptosis and autophagy, their regulation is intimately connected and the same regulators can sometimes control both apoptosis and autophagy^43^. One such regulator is the NF-κB signaling pathway. It is well known that activation of NF-κB is capable of inhibiting apoptosis^44^,^45^,^46^,^47^,^48^,^49^. NF-κB can also regulate autophagy either in a positive or a negative manner^50^,^51^,^52^,^53^. Our results suggest that phycocyanin-mediated complex interplay between autophagy and apoptosis may converge upon NF-κB activation. On one hand, phycocyanin induces autophagy by suppressing the Akt/mTOR pathway, whose activation counteracts the pro-survival effects of NF-κB activation on gene expression in response to DNA damage and cytokines^54^. mTOR deficiency or inactivation increases phosphorylation and nuclear translocation of nuclear factor NF-κB, which results in enhanced NF-κB activation^55^. Since NF-κB is one of the transcription factors for Beclin 1^24^, a key initiator of cellular autophagy, phycocyanin-induced nuclear translocation of NF-κB may play an important role during this process. The findings of this study indicate that the expression of Beclin 1 is highly correlated with the amount of NF-κB translocated into the cell nucleus. On the other hand, phycocyanin is able to induce apoptosis of PANC-1 cell by activating p38 and JNK signaling pathways and inhibiting Erk pathway. Moreover, the translocation of NF-κB activated by phycocyanin enhances cell autophagy by increasing the transcription of Beclin 1. In conclusion, our studies reveal an important signaling mechanism that phycocyanin induces apoptotic and autophagic cancer cell death via balancing the complex regulation of the MAPK, Akt/mTOR and NF-κB signaling pathways.

## Materials and Methods

### Reagents and antibodies

Phycocyanin (electrophoretic purity) was isolated and purified from *Spirulina platensis* according to the protocols reported previously with minor modifications^56^. Isolated phycocyanin was dissolved at a concentration of 1 mM in PBS (pH 7.4) as a stock solution and kept at −80 °C. It was diluted with RPMI-1640 medium (Gibco, 23400-021) before each experiment to keep the final concentration the solvent less than 5% (v/v) throughout the study.

Primary antibodies to MAP-LC3, cathepsin D, LAMP-1, p70S6K, p-p70S6K and Histone H3 were available from Santa Cruz Biotechnology (USA). Primary antibodies against procaspase, caspase, PARP and GAPDH were purchased from Beyotime Institute of Biotechnology (China). Primary antibodies for Beclin 1, Akt, p-Akt, P38, p-P38, Erk1/2, p-Erk1/2, JNK, p-JNK, mTOR, p-mTOR and NF-κB were from Cell Signaling Technology (USA). Horse radish peroxidase-conjugated secondary antibodies were purchased from sigma (USA). Fluorescence-conjugated secondary antibodies were from Invitrogen (USA). Chloroquine (Chlor) were purchased from sigma (USA). NF-κB SN50 and PD98059 were from Merck Millipore (USA). Caspase 3 siRNA, Beclin 1 siRNA and control siRNA was obtained from Cell Signaling Technology, Inc. (CST, USA).

### Cell culture

PANC-1, Capan-1, BxPC3, H460, QSG-7701 and AC-16 cells cells were cultured in RPMI 1640. HepG2, BGC-823, DU145, MCF-7 and HK-2 cells were cultured in DMEM. HUVEC was cultured in ECM Media supplemented with growth factors. NK-92 was grown in RPMI 1640 with 2 ng/mL IL-2 (R&D, USA). All the cells were purchased from ATCC. Cells were kept at 37 °C in humidified air with 5% CO_2_. Media were supplemented with penicillin G (100 U/ml), streptomycin (100 mg/ml) and 10% FBS.

### Cell viability assay

Cell viability was analyzed using the CellTiter-Glo Assay kitper the manufacturer’s instruction (Promega) as previously described^57^.

### Colony formation assay

Cells in the exponential growth phase were harvested and seeded at about 1000 cells per well in a six-well plate. After 12 h incubation, cells were treated for another 24 h with 2.5, 5, 10 and 20 μM phycocyanin, and then continuously incubated in fresh medium at 37 °C in 5% humidified CO_2_. After incubation for 10–14 days, cells were washed with PBS twice, fixed with methanol for 15 min, stained with 0.5% crystal violet for 15 min at room temperature, and were observed under an optical microscope^40^.

### Xenograft tumor

The study was approved by China Pharmaceutical University licensing committee. The experiment was performed in accordance with approved guidelines. Human pancreatic carcinoma PANC-1 cells (5 × 10^6^ cells in 100 μl of serum-free RPMI 1640 medium) were inoculated subcutaneously into the axillary fossa of the nude mice. Tumor growth was measured daily with calipers. Tumor volume was calculated as (L × W2)/2, where L is the length in millimeters, and W is the width in millimeters. When the tumors reached a mean volume of 80 to 110 mm^3^, the mice were randomly divided into five groups (each group contained six mice): 0.9% normal saline control group, 10 mg/kg, 25 mg/kg and 50 mg/kg phycocyanin groups. Phycocyanin and vehicle treatments were given peritoneal injection (i.p.) once every other day during a thirty-day experiment. Tumor size was measured once every other day. The body weight of the animals was measured twice a week while the general health status of the animals was monitored daily. At the end of treatment, all mice were sacrificed and tumors were excised and weighed.

### Caspase 3 activity assay

After treatment, PANC-1 cells were collected by centrifugation at 1000 × g and washed 3 times with phosphate buffered saline (PBS). Cell pellets were resuspended in PBS. Cell extracts were prepared by freeze/thawing cycles, followed by centrifugation for 15 minutes at 14,000 × g. All procedures were conducted at 4 °C. Protein concentrations of the supernatant fractions were measured using the Pierce BCA Protein Assay Kit (Thermo Fisher Scientific, USA). Caspase 3 activities, expressed as RFU/mg protein, were measured by Caspase-Glo^TM^ 3/7 Assay kit (Promega, USA) in 96-well plates according to the manufacturer’s instructions with a microplate reader (BMG, Germany).

### Immunofluorescence analysis

PANC-1 cells were treated with 10 μM phycocyanin for 0, 3, 6, 12, 24 and 48 h. Co-treatment with 3 MA (10 mM) or (20 μM) chloroquine were performed by pretreated the cells with 3 MA or chloroquine 2 h followed by phycocyanin treatment for 48 h. Cells were fixed with 4% paraformaldehyde in PBS at 1 h intervals, permeabilized with 0.5% Triton X-100, and blocked with 2% BSA for 30 min. Incubation with primary antibodies (diluted 1:200) against MAP-LC3 was done overnight at 4 °C. After washing, cells were exposed to FITC-conjugated antibody (1:500, Invitrogen, USA). Lysosomal-rich/acidic compartments were visualized with LysoTracker Red DND-99 (Invitrogen, L7528, USA), used at a final concentration of 25 nM and added 1 h before imaging. Lysosomal-dependent proteolysis was visualized with DQ Green BSA (Invitrogen, D12050, USA), at 10 μg/ml and added 1 h before imaging. After washing, the nuclei were stained with DAPI (Sigma, USA) 10 min before imaging. A High Content Screening system (ImageXpress^®^ Micro, Molecular Devices, USA) was used for co-localization analysis.

### Cell apoptosis assay

Prepared PANC-1 cells (1 × 10^6^/ml) were washed twice with cold PBS and then resuspended gently in 500 μl binding buffer. Thereafter, cells were stained in 5 μl Annexin V-FITC (DOJINDO, Japan). Finally, 5 μl PI (Beyotime Biotechnology, China) was added to these cells and incubated for 20 min in a dark. Stained cells were analyzed by FACS Calibur (Becton Dickinson, USA).

### Cell cycle assay

After treatment, cells were trypsinized, harvested, and fixed in 1 ml 80 % cold ethanol in test tubes and incubated at 4 °C for 15 min. Cells were then centrifuged at 1,500 rpm for 5 min and the cell pellets were resuspended in 500 μl of PI/RNase staining buffer (BD Biosciences, USA), incubated on ice for 30 min and washed twice with cold PBS. Cell cycle distribution was calculated from 10,000 cells with ModFit LTTM software (Becton Dickinson, CA, USA) using FACS caliber (Becton Dickinson, CA, USA).

### Western blot assay

Cells were washed with ice-cold PBS and lysed in buffer containing 50 mM Tris-HCl (pH 7.4), 150 mM NaCl, 1 mM EDTA, 1 mg/ml pepstatin A, 1 mM PMSF and 1% Triton X-100. Cell lysates were subjected to 10–14% SDS-PAGE and electrotransferred to PVDF membranes. After blocking, membranes were incubated at 4 °C for 12 h with the indicated primary antibodies and for 60 min with the corresponding horse radish peroxidase–conjugated secondary antibodies. Membranes were washed 3 times in Tris-buffered saline containing 0.5% Tween-20 for 5 min after each incubation step. Bound antibodies were visualized using ECL (PerkinElmer, USA).

### Plasma and Nuclear protein extraction

Whole-cell or nuclear lysates for NF-κB detection were isolated using nuclear and cytoplasmic protein extraction kit (Beyotime, China). The isolated proteins were then analyzed using immunoblotting.

### siRNA transfection

Human caspase 3 siRNA (#6466, CST, USA) and Beclin 1 siRNA (#6246, CST, USA) were used following the manufacturer’s instructions. Transfections were carried out as previously described^58^. Briefly, PANC-1 cells were plated in 96-well plates at 10,000 cells/well 24 h before transfection. Transfection was carried out by adding a 2:1 (v/w) of liposome 2000 Reagent/siRNA mixture in a final volume of 10 μL into the cultured cells. After 24 h incubation, cells were applied for various experiments.

### Statistical analysis

The statistical significance of the differences between experimental variables and their reference group was determined using the Student’s t test. *P* < 0.05 was considered statistically significant. The values shown represent the mean ± SD.

## Additional Information

**How to cite this article**: Liao, G. *et al*. Phycocyanin Inhibits Tumorigenic Potential of Pancreatic Cancer Cells: Role of Apoptosis and Autophagy. *Sci. Rep.* **6**, 34564; doi: 10.1038/srep34564 (2016).

## Supplementary Material

Supplementary Information
