# Supplementary material for: Phycocyanin Inhibits Tumorigenic Potential of Pancreatic Cancer Cells: Role of Apoptosis and Autophagy
Source: Sci Rep. 2016 Oct 3;6:34564. doi: 10.1038/srep34564 (PMC5046139; doi:10.1038/srep34564)
Supplement: Supplementary Information [file srep34564-s1.pdf]

Phycocyanin Inhibits Tumorigenic Potential of Pancreatic Cancer Cells: Role of Apoptosis and Autophagy

Gaoyong Liao[a], Bing Gao[a], Yingnv Gao[a], Xuegan Yang[a], Xiaodong Cheng[b]*, Yu Ou[a]*

[a]School of Life Science & Technology, China Pharmaceutical University, Nanjing, China; [b]Department of Integrative Biology and Pharmacology, The University of Texas Health Science Center, Houston, USA

*Correspondence to: Yu Ou, E-mail: njcpuoy@126.com; Xiaodong cheng, E-mail: xiaodong.cheng@uth.tmc.edu

Fig. S1

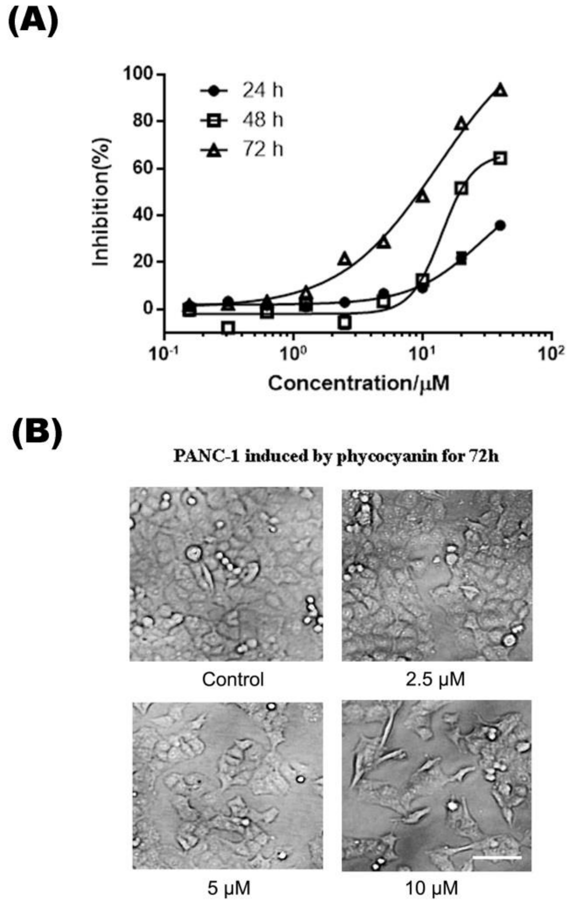

Fig. S1. Phycocyanin inhibits the tumorigenicity of PANC-1 in vitro. **(A)** Time dependent changes in the inhibition curves for PANC-1 cells treated with phycocyanin. **(B)** Effect of phycocyanin on PANC-1 cell morphology. Cells were incubated with 2.5、5 and 10 μM phycocyanin for 72 h. Cell morphology was assessed by phase contrast microscopy. Phycocyanin treated cells displayed an elongated cell morphology and increased detachment. Scale bar=100 μm.

Fig. S2

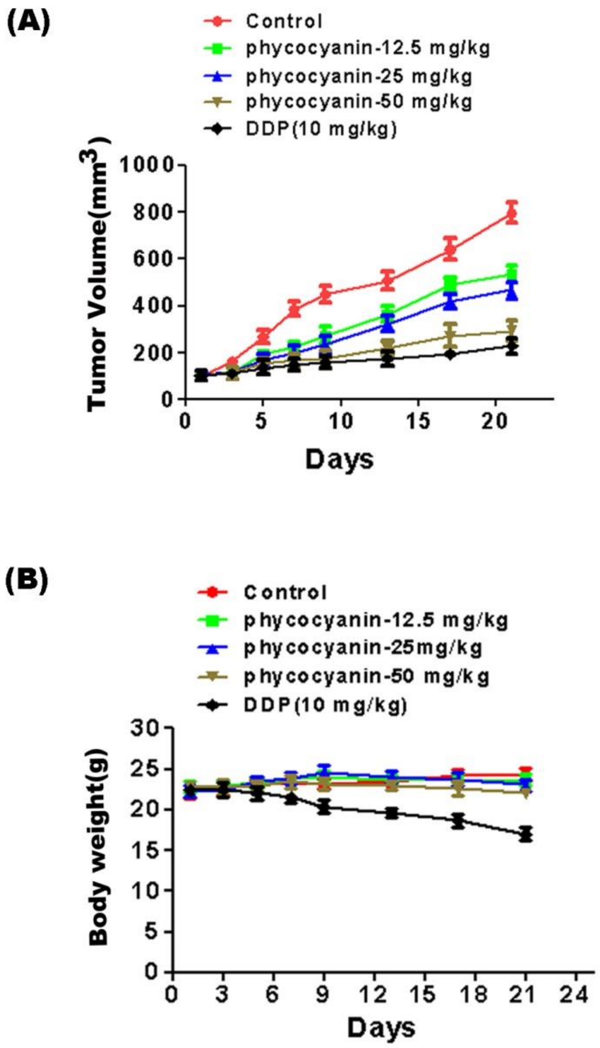

Fig. S2. Effect of Phycocyanin on the body weight and xenograft tumor weight in vivo. **(A)** Tumor volume. From the initiation of treatment, tumors were measured once every other day with calipers. Tumor volumes were calculated and data were plotted using the geometric mean for each group vs. time. Each point represented the mean tumor volume (± SD) of measurements from the six mice in each treatment group. **(B)** The body weight of nude mice for 26-day treatment.

Fig. S3

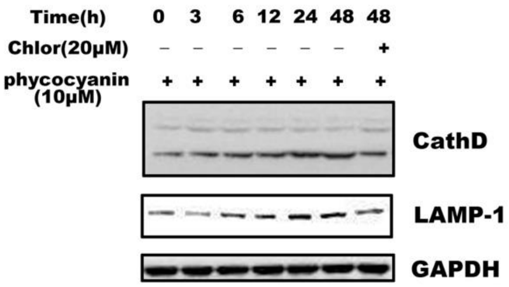

Fig. S3. Effect of Phycocyanin on the expression level of LAMP-1 and cathepsin D in vitro. Western blotting analysis of cell lysates collected for LAMP-1 and cathepsin D at the indicated time points. Cathepsin D strips showed two regions, which represented the 43–50kD forms of cathepsin D precursors and the 28 kD cathepsin D heavy chain respectively.
